# Resilience and functional redundancy of methanogenic digestion microbiome safeguard recovery of methanogenesis activity under the stress induced by microplastics

**DOI:** 10.1002/mlf2.12090

**Published:** 2023-12-15

**Authors:** Jinting Liu, Guofang Xu, Siyan Zhao, Jianzhong He

**Affiliations:** ^1^ Department of Civil and Environmental Engineering National University of Singapore Singapore

**Keywords:** functional redundancy, methanogenic digestion, microbial ecology, microplastics, resilience

## Abstract

Microplastics and nanoplastics are emerging pollutants that substantially influence biological element cycling in natural ecosystems. Plastics are also prevalent in sewage, and they accumulate in waste‐activated sludge (WAS). However, the impacts of plastics on the methanogenic digestion of WAS and the underpinning microbiome remain underexplored, particularly during long‐term operation. In this study, we found that short‐term exposure to individual microplastics and nanoplastics (polyethylene, polyvinyl chloride, polystyrene, and polylactic acid) at a low concentration (10 particles/g sludge) slightly enhanced methanogenesis by 2.1%−9.0%, whereas higher levels (30−200 particles/g sludge) suppressed methanogenesis by 15.2%−30.1%. Notably, the coexistence of multiple plastics, particularly at low concentrations, showed synergistic suppression of methanogenesis. Unexpectedly, methanogenesis activity completely recovered after long‐term exposure to plastics, despite obvious suppression of methanogenesis by initial plastic exposure. The inhibition of methanogenesis by plastics could be attributed to the stimulated generation of reactive oxygen species. The stress induced by plastics dramatically decreased the relative abundance of methanogens but showed marginal influence on putative hydrolytic and fermentation populations. Nonetheless, the digestion sludge microbiome exhibited resilience and functional redundancy, contributing to the recovery of methanogenesis during the long‐term operation of digesters. Plastics also increased the complexity, modularity, and negative interaction ratios of digestion sludge microbiome networks, but their influence on community assembly varied. Interestingly, a unique plastisphere was observed, the networks and assembly of which were distinct from the sludge microbiome. Collectively, the comprehensive evaluation of the influence of microplastics and nanoplastics on methanogenic digestion, together with the novel ecological insights, contribute to better understanding and manipulating this engineered ecosystem in the face of increasing plastic pollution.

## Impact statement

Methanogenic digestion is widely applied to recover energy from waste‐activated sludge (WAS) and decreases its harm to public health. Microplastics and nanoplastics are emerging pollutants that may interfere with ecosystem functions. However, a comprehensive understanding of the impacts of plastics on the methanogenic digestion of WAS and the underpinning microbiome has not been achieved. Our study systematically investigated the short‐ and long‐term influence of microplastics and nanoplastics on methanogenic digestion and reported the long‐term recovery behavior of methanogenesis activity for the first time. Novel ecological insights into the influence of plastics on the digestion microbiome were also provided. Our findings suggest that plastics can be an important factor in diagnosing and manipulating digester performance and microbiome.

## INTRODUCTION

Plastics have been used in massive amounts for several decades, and they are ubiquitous in the environment, causing global concerns^1^, ^2^. Conventional petroleum‐based plastics are resistant to environmental degradation; nonetheless, they tend to be fragmented into microplastics with a diameter of 1 µm−5 mm. Nanoplastics (diameter <1 µm) could be further formed during long‐term environmental weathering and aging of microplastics^3^. This exaggerates the adverse effects of plastics due to the higher toxicity of microplastics and nanoplastics than that of plastics with a larger size^4^. Wastewater treatment plants have been identified as a hotspot sink of microplastics and nanoplastics due to the high content of plastics in influent sewage^5^. Most microplastics in influent sewage cannot be effectively degraded during biological sewage treatment and are generally accumulated in waste‐activated sludge (WAS) and digestion sludge (DS) in high concentrations (38−287 particles/g dry sludge)^6^. Among these accumulated microplastics, polyethylene (PE), polyvinyl chloride (PVC), polystyrene (PS), polypropylene (PP), and polyethylene terephthalate (PET) are the dominant ones in WAS^7^.

Anaerobic digestion is widely applied to remove pollutants (e.g., pathogens and organic pollutants) in WAS and harvest renewable energy via biogas production^8^. Successful methanogenic digestion of WAS accounts for a collection of microbes that catalyze hydrolysis, acidogenesis, and methanogenesis^9^. Despite the fact that microplastics and nanoplastics are prevalent in WAS, we are at the nascent stage of understanding their impacts on subsequent methanogenic digestion and the underpinning microbial communities. Recent studies reported varying influences of microplastics on the performance of methanogenic digestion, which likely depended on the types, sizes, and concentrations of microplastics as well as DS microbiome^10^, ^11^, ^12^. Contrarily, nanoplastics consistently inhibited methanogenic digestion due to damage to cell membranes by reactive oxygen species (ROS) induced by nanoplastics^13^. These studies provided pioneering insights into the influence of an individual type of plastics on methanogenic digestion; however, a comprehensive comparison of the influence of different types of intensively used plastics on methanogenic digestion of WAS is absent. Additionally, it remains unknown whether the coexistence of multiple microplastics and nanoplastics could cause synergistic or antagonistic effects on methanogenic digestion. Complicated microbial systems usually exhibit certain levels of persistence or resilience to external disturbance and stress; namely, the microbiota could maintain its composition and functions or gradually recover to its original status when encountering disturbance^14^, ^15^. It awaits investigation on whether the performance and compositions of methanogenic digestion microbiota could recover to their original states or transit to alternative states during long‐term exposure to microplastics and nanoplastics.

Knowledge about the assembly, interactions, and stability of microbial communities in anaerobic digesters is critical to better understand, predict, and manipulate the relevant microbiota for improved digestion performance^16^, ^17^. Digestion microbiome is usually shaped by both stochastic processes (random birth‐death events, ecological drift, and probabilistic dispersal) and deterministic processes (environmental filtering and biological interactions)^17^. Deterministic processes were reported to play governing roles in the assembly of microbial communities in methanogenic digesters^18^, ^19^. However, the impacts of microplastics and nanoplastics on community assembly in methanogenic digesters are underexplored. Microplastics and nanoplastics can stimulate the production of ROS^20^, which may exhibit both deterministic (e.g., selective proliferation of specific microbial populations on plastics and environmental filtering)^21^, ^22^, ^23^ and stochastic (e.g., random killing of microbial cells) effects. Therefore, we hypothesize that the overall influence of microplastics and nanoplastics on methanogenic digestion community assembly is likely determined by the tradeoff between the changes of two assembly processes, which awaits further experimental validation. In addition, microbial networks and their stability have been proven to be closely linked with functions of ecosystems (e.g., cycling of nitrogen)^24^, ^25^. However, the changes in microbial networks and their stability in response to microplastics and nanoplastics have not been studied in methanogenic digesters treating WAS.

This study aims to comprehensively determine the short‐ and long‐term influence of different commercially available microplastics and nanoplastics on the performance of methanogenic digesters treating WAS. Succession of the digester microbiome was monitored during the long‐term operation of digesters. Particularly, the resilience of digester performance and microbiome were evaluated. Furthermore, the changes in microbial community assembly, co‐occurrence networks, and their stability in response to microplastic and nano‐plastic exposure were analyzed. The findings from these aspects are of fundamental importance to understanding and manipulating methanogenic microbiota for improved performance when facing plastic pollution.

## RESULTS

### Short‐term effects of microplastics and nanoplastics on methanogenic digestion of WAS in batch digesters

**Figure 1:**
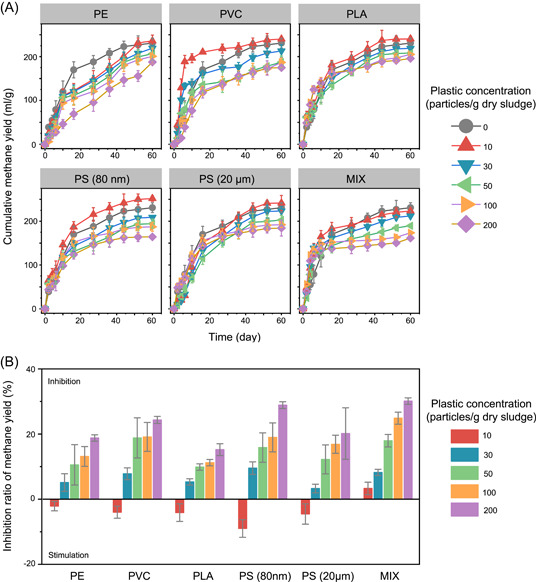
Short‐term inhibition effects of microplastics and nanoplastics on methane production in batch anaerobic digesters treating waste‐activated sludge. (A) Cumulative methane yield after 60‐day incubation. (B) Inhibition ratio of methane production after 60‐day incubation. MIX, a defined plastic mixture containing equal amounts of PE, PVC, PLA, and PS (the same for other figures unless stated otherwise); PE, polyethylene; PVC, polyvinyl chloride; PLA, polylactic acid; PS, polystyrene.

To comprehensively determine the short‐term influence of microplastics and nanoplastics on the methanogenic digestion of WAS (Table S1), batch methanogenic digesters treating WAS were spiked with different concentrations of plastics and operated for 60 days. The cumulative amounts of methane generated in all batch digesters gradually increased and reached the highest levels after 60 days of incubation (Figure 1A). The short‐term influence of microplastics and nanoplastics on methanogenic digestion largely depended on their concentrations. At the end of incubation, the cumulative amounts of methane generated were 2.1%−9.0% higher in digesters spiked with individual plastics (10 particles/g dry sludge) than that in the control digesters (Figure 1B). The enhanced methane production by low concentration of plastics could be likely attributed to enhanced solubilization of WAS^11^. However, higher concentrations of plastics suppressed methane production, and the extent of inhibition on methane production was positively correlated with the concentrations of plastics (Figure 1B). When exposed to 200 particles/g sludge, the cumulative methane yield was considerably inhibited by 15.2%−30.1%. The size of plastics also affected the short‐term influence of plastics on the methanogenic digestion of WAS, with nano‐PS exhibiting more profound inhibition than micro‐PS (Figure 1B). Measurement of ROS showed that microplastics and nanoplastics stimulated ROS production in batch digesters (Figure S1), and the concentration of ROS was positively correlated with the concentration of plastics (*r* = 0.55, *p* < 0.001). Moreover, the ROS concentration was increased by 221 ± 19%, 129 ± 19%, 78 ± 11%, 61 ± 10%, 51 ± 3%, and 46 ± 6% in batch digesters amended with PS (80 nm), MIX (a defined plastic mixture containing equal amounts of the five types of plastics), PS (20 μm), PE, PLA, and PVC at a concentration of 200 plastic/g sludge, respectively (Figure S1). The massive production of ROS may damage the metabolic activity of microbial cells, consequently inhibiting methanogenesis. Additionally, the mixture of different plastics showed synergistic inhibition on methane production, as supported by the decreased methane generation in batch anaerobic digesters spiked with the plastic mixture at a total concentration of 10 particles/g sludge but stimulated methane production in digesters amended with individual plastics at the same concentrations (Figure 1B). These results elucidated that environmentally relevant high concentrations (30−200 particles/g sludge) of microplastics and nanoplastics consistently inhibited methane production during short‐term methanogenic digestion of WAS, although the extent of inhibition was affected by the sizes, types, and concentrations of plastics.

### Adaption and recovery of methanogenesis activity during long‐term exposure to microplastics and nanoplastics

**Figure 2:**
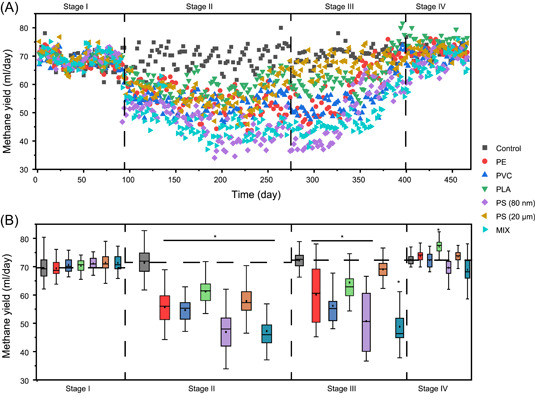
Recovery of semicontinuous digester performance of methanogenic digesters treating waste‐activated sludge during long‐term operation with amendment of different plastics. (A) Daily methane yield in methanogenic digesters in different operation stages. (B) Comparison of methane yield in methanogenic digesters in different operation stages. Stage I, Days 1−90, no plastic amendment; stage II, Days 91−225, performance inhibition; stage III, Days 225−400, gradual performance recovery; stage IV, Days 400−466, stable performance. Asterisks indicate a statistically significant difference (*p* < 0.05) in methane production in plastic‐exposed digesters compared to the control digesters without plastic amendment.

The long‐term influence of microplastics and nanoplastics on the methanogenic digestion of WAS was further investigated in semicontinuous anaerobic digesters (sluge retention time, 24 days), which were operated for 466 days. Interestingly, the operation of anaerobic digesters could be divided into four stages based on digestion performance. In stage I (Days 1−90; no plastic amendment), all digesters were successfully started up with comparable methane production (127.2 ± 5.7 ml/day; Figure 2A). However, amendment of either individual or mixture of microplastics and nanoplastics dramatically decreased methane production in stage II (Days 91−225). The methane yield was inhibited by as much as 21.3 ± 7.6%, 22.7 ± 5.6%, 13.8 ± 7.6%, 33.3 ± 7.6%, 18.4 ± 7.1%, and 32.8 ± 8.4% in digesters spiked with PE, PVC, PLA, PS (80 nm), PS (20 μm), and MIX at 200 particles/g sludge, respectively, compared to the control digesters without plastic amendment (Figure 2B). This was consistent with the inhibitory effects of high concentrations of microplastics and nanoplastics on methanogenic digestion observed in batch experiments. Unexpectedly, we observed that methane yield in plastic‐exposed digesters gradually recovered in stage III (Days 225−400) and reached the highest levels in stage IV (Days 400−466), which was comparable to the methane yield in the control digesters (Figure 2A). Correspondingly, the dissolved organic carbon (DOC) removal rate was comparable in all digesters in stage I (78.6 ± 1.6%), but it was significantly lower in plastic‐exposed digesters during stages II/III (65.7 ± 4.1% and 67.4 ± 6.6%, respectively) and returned to a similar level in stage IV (76.8 ± 3.7%; Figure S2). These observations demonstrate that methanogenic digestion microbiota could adapt to the stress induced by microplastics and nanoplastics and recover their metabolic activities after long‐term operation (>300 days). Despite the good recovery of methanogenesis activity, the concentrations of ROS in semi‐continuous digesters spiked with plastics were still significantly higher than that in the control digester at stage IV (Figure S3), suggesting that the digestion microbiome may have adapted to ROS.

### Resilience and functional redundancy of DS microbiome

**Figure 3:**
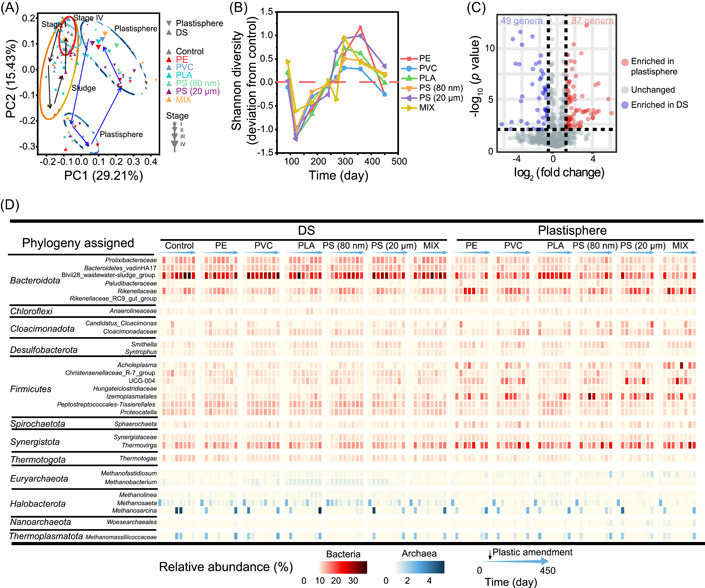
Resilience and functional redundancy of methanogenic digestion microbiome in semicontinuous digesters amended with different plastics. (A) Succession of dominant microbial lineages in digestion sludge (DS) and the plastisphere. (B) Recovery of microbial community structure in DS and plastisphere as revealed by principal coordinates analysis (PCoA). (C) Changes in the difference of Shannon diversity between digesters encountering plastic stress and the control without plastic exposure. (D) Unique plastisphere microbiome exhibiting differential abundance of microbial populations.

Methanogenesis in anaerobic digesters is closely linked with the digestion microbiome. The recovery of methanogenesis activity in digesters during long‐term exposure to plastics indicates that the digestion microbiome is resilient to plastic‐induced stress, or/and it has high functional redundancy so that different functionally similar microbial populations could take over the roles of suppressed microbes. To test this hypothesis, the microbiome succession in methanogenic digesters exposed to different plastics was monitored and analyzed. Principal coordinates analysis (PCoA) showed that microbial communities of DS in all methanogenic digesters were closely clustered together in stage I (Figure 3A), suggesting similar microbial community structures. Microbial communities in plastic‐exposed digesters diverged from those in the control digesters in stage II when different plastics were amended to digesters, nonetheless, which gradually converged in stage III despite continuous exposure to plastics. In the end, the microbiome in digesters spiked with plastics returned to be similar to that in the control digesters, as supported by the close clustering of microbial communities of all digesters in the PCoA plot in stage IV. The changes in microbial diversity also revealed the high resilience of the DS microbiome. The diversity of microbial communities was similar in all digesters at the end of stage I, but it was less diverse in plastic‐exposed digesters in stage II (Figure 3B). Nonetheless, it rebounded back to digesters receiving plastics in stage III and stabilized to be similar to the control digesters at the end of stage IV. These results demonstrated the resilience of the sludge microbiome in methanogenic digesters treating WAS in response to plastic‐induced stressors, in line with the recovery of methanogenesis activity during continuous exposure to plastics.

In addition to community resilience, functional redundancy potentially contributes to the recovery of methanogenesis activity in digesters under plastic stress. This was well demonstrated by the digester‐specific succession of distinct methanogenic populations in different operation stages (Figure 3C). Multiple methanogens (e.g., *Methanosaeta*, *Methanofastidiosum*, *Methanobacterium*, and *Methanolinea*) were detected during the start‐up of digesters in stage I, which served as an initial pool of functionally redundant species for selection by plastic‐induced stressors. *Methanosaeta* was the dominant methanogen in most samples from the control digesters, although the relative abundance of which varied with operation time (Figure 3C). However, the initially abundant *Methanosaeta* was replaced by *Methanosarcina* and *Methanomassiliicoccaceae* in stage IV in digesters exposed to PE, PVC, PLA, PS (20 μm), and MIX, whereas *Methanosaeta* and *Methanobacterium* were retained as the predominant methanogens in digesters amended with nano‐PS (80 nm). In contrast, microplastics and nanoplastics exhibited comparatively lower influence on putative hydrolytic and acidogenesis populations. For example, *Leptolinea*, together with *Caldilineaceae*, co‐existed as potential hydrolytic populations; Blvii28_wastewater‐sludge_group and *Peptostreptococcales‐Tissierellales* were the dominant putative microbes that have been verified with metabolic capability to produce short‐chain fatty acids throughout 466 days operation of digesters exposed to different plastics. These observations showed that hydrolytic and acidogenic populations in methanogenic digesters are more resistant to plastic‐induced stress than methanogens; nonetheless, functional redundancy of methanogens can safeguard the recovery of methanogenic activity during long‐term plastic exposure.

### Plastisphere microbiome is distinct from the sludge microbiome

Plastic surfaces have been identified as a unique niche for selective colonization and growth of specific microorganisms (defined as the plastisphere), which are often distinct from the microbiome in surrounding environments in a variety of natural ecosystems^22^. In this study, we also compared the microbiome on plastics with the sludge microbiome in methanogenic digesters to confirm whether a unique plastisphere could be established in this engineered system. Results showed that the plastic microbiome was well separated from the sludge microbiome in the PCoA plot, forming distinct clusters (Figure 3A). The PERMANOVA analysis further revealed a statistically significant difference (*p* < 0.05) between the plastic microbiome and the DS microbiome (Table S2). These analyses validate that a unique plastisphere microbiome was established in methanogenic digesters treating WAS. Additionally, the plastisphere in digesters also showed obvious succession in a similar manner as that of the sludge microbiome; that is, the plastisphere microbiome initially diverged in the initial days (i.e., stage II) of plastic exposure and gradually converged after long time adaption to plastics in stage III and IV (Figure 3A). Detailed analysis of the relative abundance of each taxon revealed selective proliferation of 87 genera in the plastisphere, whereas 49 genera tended to grow with higher relative abundance in DS (Figure 3C). Specifically, *Clostridium_sensu_stricto_18, Proteiniphilum*, *Lentimonas*, *Desulforhabdus*, *Pseudoxanthomonas*, and *Bellilinea* were detected at significantly higher abundance in the plastisphere than that in DS. Additionally, the relative abundance of putative pathogens (*Escherichia‐Shigella*, *Parvimonas*, and *Acholeplasma*) and organohalide‐respiring bacteria (OHRB), including *Dehalobacter* and uncultured *Dehalococcoidia*, were significantly higher on the plastisphere compared with that in sludge, consistent with the reported accumulation of pathogens and OHRB in the plastisphere^20^, ^26^, ^27^. Altogether, a unique plastisphere is proved to be formed in methanogenic digesters treating WAS.

### Plastics alter co‐occurrence networks, stability, and assembly of digestion microbiome

**Figure 4:**
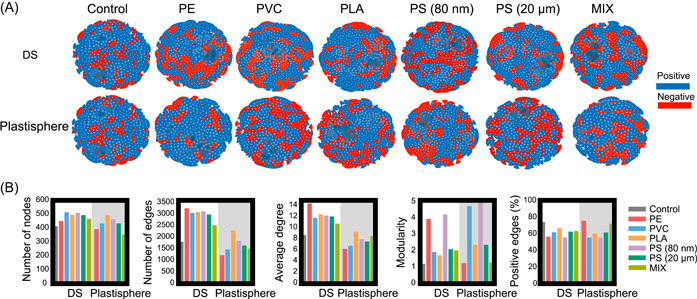
Plastics intensify co‐occurrence networks of DS with higher stability in semicontinuous digesters treating waste‐activated DS. (A) Microbial co‐occurrence networks in DS and plastisphere microbiome. (B) The topological properties in DS and plastisphere microbiome.

Understanding interspecies interactions and assembly of microbial communities are two central tasks in microbial ecology, which is indispensable to understanding and manipulating the microbiome to control the catalyzed processes. Therefore, we performed co‐occurrence network and assembly analyses of microbial communities inferred from 16 S rRNA gene sequencing to gain more ecological insights into the impacts of microplastics and nanoplastics on the methanogenic digestion microbiome. For the DS microbiome, the amendment of plastics consistently increased the overall complexity of networks compared to the control digesters, as supported by more nodes and edges and a higher average degree of networks in digesters exposed to plastics (Figure 4 and Table S3). The intensified co‐occurrence networks might contribute to alleviating inhibition caused by plastic‐induced stress. Notably, higher modularity and more negative edges were also observed in co‐occurrence networks of sludge microbiome in digesters exposed to plastics than the control digesters, suggesting higher stability of networks in the former^28^. In contrast, the complexity of networks in the plastisphere was comparatively lower than that in the DS, with substantially fewer nodes and edges in the former (Figure 4 and Table S3). Collectively, microplastics and nanoplastics could significantly improve community stability by increasing network complexity, modularity, and negative interactions in methanogenic digesters.

**Figure 5:**
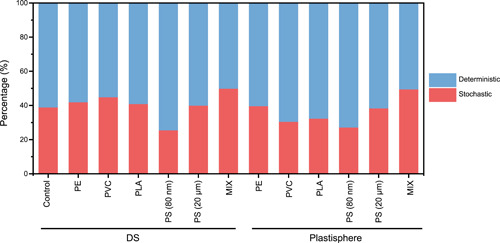
Dominance of deterministic processes in assembling DS and plastisphere microbiome in semicontinuous digesters exposed to plastics.

Microbial community assembly is usually governed by both stochastic and deterministic processes, which can be quantified by the normalized stochasticity (NST)^29^. In all the methanogenic digesters, the NST values were consistently lower than 50%, implicating the governing roles of deterministic processes in community assembly (Figure 5). Interestingly, amendment of the defined plastic mixture (MIX) significantly increased NST of sludge microbiome from 39.2 ± 7.9% in the control digesters to 50.1 ± 7.7%, whereas other microplastics (PE, PVC, PLA, and PS) did not obviously affect the NST values of sludge microbiome in digesters. Contrarily, exposure to nanoplastic PS (80 nm) drastically decreased the NST value of the DS microbiome to 25.8 ± 11.1%. Compared to the DS microbiome, the NST values of the plastisphere microbiome (35.5 ± 8.0%) were much lower at a statistically significant level (*p* < 0.05); this is consistent with the notion that microbial colonization on plastics is highly selective and deterministic. These observations suggest that exposure to individual microplastics only caused marginal influence on DS community assembly, but nanoplastic alone and plastic mixture containing nanoplastic could dramatically change community assembly despite in distinct directions.

## DISCUSSION

Microplastics and nanoplastics are emerging contaminants in wastewater treatment plants, most of which are accumulated in WAS during sewage treatment. This is believed to have a substantial influence on subsequent methanogenic digestion of WAS because increasing evidence suggests that microplastics and nanoplastics considerably affect the biological cycling of carbon, nitrogen, and sulfur in diverse natural ecosystems^30^, ^31^. Recently, it was reported that short‐term exposure to single microplastics (40 µm polyethylene and 1 mm polyvinyl chloride) at high concentrations severely inhibited methanogenic digestion of WAS, whereas lower concentrations of these plastics either showed marginal or stimulatory effects in two separate studies^10^, ^11^. Notably, the threshold concentrations of these microplastics to cause inhibition on methanogenesis varied considerably in the range of 10−60 particles/g dry sludge in these previous studies^10^, ^11^, which could be likely attributed to differences of microplastic sizes or/and DS microbiome. These uncontrolled factors prevent us from a comprehensive and fair comparison of the influence of different microplastics on the methanogenic digestion of WAS. In this study, the discovery that nanoplastics (80 nm) inhibited methanogenesis more severely than microplastics made of the same material and at the same concentration is consistent with the reported higher toxicity of nanoplastics to microbial cells^20^. More importantly, the coexistence of different microplastics and nanoplastics exhibited synergistic effects, particularly at low concentrations (i.e., 10 particles/g sludge) for more extensive inhibition on methanogenesis, implying that the short‐term inhibitory effects of microplastics and nanoplastics on methanogenic digestion of WAS are likely underestimated due to the ubiquitous coexistence of multiple types of plastics in WAS^32^. In this study, all the tested microplastics and nanoplastics stimulated production of ROS in digesters as well as previous reports^20^, ^33^, suggesting that ROS production is likely a universal mechanism underlying the observed inhibition on methanogenesis. The increased production of ROS may more severely interfere with the antioxidant system in microbial cells^34^, ^35^, suppressing cell growth and damaging cell membrane^36^, ^37^. Along with higher levels of ROS in digesters exposed to plastics, the concentrations of extracellular polymeric substances (EPS) significantly decreased (Figure S4), which may cause both stimulatory and inhibitory effects on the methanogenic digestion of WAS. EPS plays crucial roles in protecting the buried microbial cells against environmental stress, therefore, microbial cells with less surrounding EPS might be more susceptible to ROS inactivation^13^. On the other hand, the disintegration of EPS and microbial cells has been reported as a critical step to increase the digestibility of WAS^38^. The overall influence of ROS induced by plastics is likely determined by the trade‐off between the stimulatory and inhibitory effects.

The functions of complex systems usually exhibit certain levels of resilience to disturbance and stress; that is, the functions could recover after adaption to the stress^39^. The stability of an anaerobic digestion system relies on the intricate syntrophic interactions between microorganisms involved in various functional components, such as hydrolysis, acetogenesis, and methanogenesis^9^. DS harbors a flexible community structure, where multiple populations may carry out similar functions, thus ensuring increased process stability through redundancy. Methanogenesis performance in digesters has been reported to partially or completely recover after long‐term exposure to stressors, such as high free ammonia concentration, increased organic loading, and high salinity due to functional redundance^40^, ^41^, ^42^. In this study, the long‐term impacts of microplastics and nanoplastics on the methanogenic digestion of WAS were monitored for over 400 days, revealing the complete recovery of methanogenesis activity under continuous exposure to plastics for the first time. This finding enlightens us to re‐evaluate the influence of plastics on the performance of methanogenic digesters treating WAS, particularly during long‐term operations. Furthermore, resilience and functional redundancy might contribute to the functional stability during severe disturbances caused by plastic amendment, despite requiring a sufficient operating time for adaptation^43^. Notably, fermentation populations seem to be resistant to plastic‐induced stress, with similar predominant fermenters being observed in different digesters^44^, ^45^. Methanogens are comparatively more susceptible to the stress caused by plastics; nonetheless, the coexistence of multiple functionally redundant methanogenic populations in the initial DS safeguards the recovery of methanogenesis activity, with the emergence of distinct methanogens in digesters amended with different plastics at the end of operation. This observation suggests that the initial digestion microbiome (e.g., diversity and composition of functional populations) might be an important factor determining the recovering behavior of digester performance under stress such as plastics, which warrants further investigation in future studies.

Deciphering interspecies interactions and the assembly of microbial communities is an essential step in manipulating the microbiome for predictable and desirable performance. The observation that microplastics and the defined plastic mixture tended to shape the assembly of DS microbiome to be more stochastic, whereas nanoplastic increased the contribution of the deterministic process to community assembly, contradicts our expectation that all microplastics and nanoplastics should change community assembly to be more deterministic due to the higher stress caused by increasing levels of ROS. Recent studies indicated that elevated and high levels of oxidative stress can lead to a more deterministic community assembly, whereas moderate or low levels of stress may favor stochastic processes^46^. One  plausible explanation for this discrepancy is that ROS at the concentrations induced by microplastics may increase stochastic processes (e.g., random killing of microbial cells by ROS) at higher extents than the deterministic process (e.g., limited environmental filtering), consequently leading to more stochastic assembly. However, the significantly higher levels of ROS generated in digesters spiked with nanoplastic may pose higher environmental filtering and shape community assembly to be more deterministic. Additionally, the assembly of the plastisphere is more deterministic compared to that of the DS microbiome, in line with the notion that microbial colonization on plastics is a selective process that depends on both the properties of plastics and microbial species^47^. Collectively, this study contributes as the first comprehensive evaluation of the influence of environmentally relevant microplastics and nanoplastics on methanogenesis performance and the underpinning microbiome in digesters treating WAS, particularly during long‐term operation. The novel mechanistic and microbial ecological insights gained in this study contribute to better understanding, predicting, and manipulating methanogenic digestion microbiome for improved performance with increasing plastic pollution.

## MATERIALS AND METHODS

### Source of sludge and microplastics

DS was collected from an anaerobic digester in a domestic wastewater treatment plant in Singapore as the inoculum to start laboratory anaerobic digesters. Fresh WAS collected from aeration tanks in the same wastewater treatment plant was concentrated to approximately 35 g/l (dry weight) and pretreated at 170°C for 70 min as the influent substrate for laboratory digesters^48^. The characteristics of DS, WAS, and thermal pretreated WAS are provided in Table S1. The background concentrations of microplastics in WAS and DS were 1.6 ± 0.3 and 1.2 ± 0.2 particles/g dry sludge (Figure S5). PE, PVC, and PLA were purchased from Sigma‐Aldrich with a diameter of 20−40 μm. PS with a diameter of 80 nm and 20 μm was purchased from Tianjin BaseLine ChromTech Research Centre.

### Setup and operation of methanogenic digesters

Batch methanogenic digesters were set up by inoculating 80 ml DS and 20 ml pretreated WAS to investigate the influence of different concentrations of microplastics and nanoplastics on the methanogenic digestion of WAS. PE, PVC, PLA, PS (20 μm and 80 nm), and a defined plastic mixture (MIX, containing equal amounts of the five types of plastics) were amended to batch digesters at final concentrations of 10, 30, 50, 100, and 200 particles/g dry weight sludge (Figure S6), which are within the concentrations of plastics detected in the environment^6^. Batch digesters without additional amendment of plastics were set as the controls. All batch digesters were set up with triplicates and incubated at 35 ± 1°C in a shaker (120 rpm).

To investigate the long‐term effects of microplastics and nanoplastics on methanogenic digestion and the response of associated microbial communities, another set of methanogenic digesters was established and operated in a semicontinuous mode for 466 days with an sluge retention time of 24 days. About 160 ml DS and 40 ml pretreated WAS were inoculated into the semicontinuous digesters with a volume of 250 ml. All digesters were stably operated in stage I for 90 days with comparable digestion performance. Microplastics and nanoplastics were then spiked into the digesters with a concentration of 200 particles/g dry‐weight sludge (i.e., the weight of WAS and DS), and a continuous plastic amendment was carried out during sludge replacement to maintain a stable concentration of plastics. The digesters were set up with duplicates and operated at 35 ± 1°C and 120 rpm for 466 days.

### DNA extraction, PCR, and amplicon sequencing

Sludge cells were harvested from 1.5 ml homogenized sludge samples by centrifugation (15,000*g*, 15 min, and 4°C) for genomic DNA (gDNA) extraction. Sludge containing PVC was filtered with a 20 μm metal mesh to obtain the high‐density plastics and then washed with deionized water three times to remove any loosely attached cells. The floated supernatant containing plastics (as the density of other plastics was lower than sludge liquid) was transferred to 1.5 ml deionized water and washed three times, followed by centrifugation (11,000*g*, 1 min, and 4°C) to harvest plastics with tightly attached biofilm. The extracted plastic samples were subjected to microscopic analysis to confirm the presence of substantial quantities of plastics (Figure S7). The collected sludge and plastisphere samples were preserved as previously described^49^. The gDNA was extracted using the QIAGEN DNeasy PowerSoil Pro Kit (QIAGEN), and the V4−V5 regions of the 16S rRNA genes of gDNA were amplified using the U515F/U909R primers as previously described^50^. Amplicon sequencing was conducted on an Illumina NovaSeq. 6000 platform (Illumina) in Novogene AIT (Singapore) and the sequencing data were analyzed using QIIME2 v2021.4.0^51^. Quality filtering, denoising, chimera removal, and identification of amplicon sequence variants (ASVs) were performed using DADA2^52^. ASV alignment and phylogeny construction were accomplished using MAFFT and RAxML, respectively^53^, ^54^. Taxonomy was assigned to ASVs against the Silva138 database by a naïve Bayes classifier (q2‐feature‐classifier classify‐sklearn)^55^, ^56^. Microbial diversity was assessed using the q2‐diversity plugin. To evaluate differences in microbial communities, adonis Permutational ANOVA (PERMANOVA) based on the Bray–Curtis metric was performed.

### Community assembly analysis and construction of co‐occurrence networks

To estimate the contribution of stochastic and deterministic processes to community assembly, the normalized stochasticity ratio (NST) was estimated using the NST package as previously reported^57^. The sludge microbiome and plastic microbial communities were grouped based on the types of plastics amended to the digesters (eight samples/group) to reveal the influence of plastics on community assembly. Co‐occurrence networks were also separately constructed for microbial communities in sludge and plastics from semicontinuous digesters spiked with different plastics, with eight samples being included for each network analysis. All network analyses were performed using the R packages VEGAN (v2.5‐7, https://github.com/vegandevs/vegan), igraph (v1.2.6, https://r.igraph.org/), and Hmisc (v4.5‐0, https://hbiostat.org/R/Hmisc/). Correlations among different microbial populations were evaluated using Spearman's correlation coefficient. The correlations with a correlation coefficient >|0.6| and *p* < 0.01 were retained for further analysis. The Benjamini–Hochberg method was used to adjust *p*‐values to reduce false‐positive correlations. Gephi (v0.9.2) was used for the visualization of networks^58^.

### Analytical methods

Approximately 15 ml of homogenized sludge from digesters was centrifugated at 5800*g* for 15 min, and the supernatant was filtered with a 0.45 µm filter for subsequent analyses. The concentration of DOC was measured using a TOC analyzer (TOC‐VCSH) with a combustion catalytic oxidation temperature of 680°C. A gas chromatograph (Agilent 7890B) equipped with a thermal conductivity detector using nitrogen as the carrier gas was used to detect biogas composition. The sludge properties, including total suspended solid and volatile suspended solid (VSS), were measured according to reported methods^59^. More detailed information about the measurement of ROS, extraction of EPS, and calculation of methane gas production is provided in Supporting Information Material.

## AUTHOR CONTRIBUTIONS

**Jinting Liu**: Conceptualization (equal); investigation (equal); validation (equal); visualization (equal); writing—original draft (equal). **Guofang Xu**: Supervision (equal); visualization (equal); writing—original draft (equal); writing—review and editing (equal). **Siyan Zhao**: Supervision (equal); writing—review and editing (equal). **Jianzhong He**: Conceptualization (equal); funding acquisition (lead); supervision (equal); writing—review and editing (equal).

## ETHICS STATEMENT

There was no animal or human experiment involved in this study.

## CONFLICT OF INTERESTS

The authors declare no conflict of interests.

## Supporting information

Supporting information.

## Data Availability

Raw Illumina Novaseq sequencing reads were deposited to NCBI with an accession number of PRJNA974881.

## References

[1] Cózar A , Echevarría F , González‐Gordillo JI , Irigoien X , Úbeda B , Hernández‐León S , et al. Plastic debris in the open ocean. Proc Natl Acad Sci USA. 2014;111:10239–10244. 24982135 10.1073/pnas.1314705111 PMC4104848

[2] Evangeliou N , Grythe H , Klimont Z , Heyes C , Eckhardt S , Lopez‐Aparicio S , et al. Atmospheric transport is a major pathway of microplastics to remote regions. Nat Commun. 2020;11:3381. 32665541 10.1038/s41467-020-17201-9 PMC7360784

[3] Cole M , Lindeque P , Halsband C , Galloway TS . Microplastics as contaminants in the marine environment: a review. Marine Poll Bull. 2011;62:2588–2597. 10.1016/j.marpolbul.2011.09.025 22001295

[4] Yong C , Valiyaveettil S , Tang B . Toxicity of microplastics and nanoplastics in mammalian systems. Int J Environ Res Public Health. 2020;17:1509. 32111046 10.3390/ijerph17051509 PMC7084551

[5] Cheung PK , Fok L . Characterisation of plastic microbeads in facial scrubs and their estimated emissions in Mainland China. Water Res. 2017;122:53–61. 28591661 10.1016/j.watres.2017.05.053

[6] Harley‐Nyang D , Memon FA , Jones N , Galloway T . Investigation and analysis of microplastics in sewage sludge and biosolids: a case study from one wastewater treatment works in the UK. Sci Total Environ. 2022;823:153735. 35149057 10.1016/j.scitotenv.2022.153735

[7] Alimi OS , Farner Budarz J , Hernandez LM , Tufenkji N . Microplastics and nanoplastics in aquatic environments: aggregation, deposition, and enhanced contaminant transport. Environ Sci Technol. 2018;52:1704–1724. 29265806 10.1021/acs.est.7b05559

[8] Ge H , Jensen PD , Batstone DJ . Pre‐treatment mechanisms during thermophilic–mesophilic temperature phased anaerobic digestion of primary sludge. Water Res. 2010;44:123–130. 19800093 10.1016/j.watres.2009.09.005

[9] Wu Y , Liu X , Wang D , Chen Y , Yang Q , Chen Y , et al. Iron electrodes activating persulfate enhances acetic acid production from waste‐activated sludge. Chem Eng J. 2020;390:124580.

[10] Wei W , Huang Q‐S , Sun J , Dai X , Ni B‐J . Revealing the mechanisms of polyethylene microplastics affecting anaerobic digestion of waste activated sludge. Environ Sci Technol. 2019;53:9604–9613. 31335125 10.1021/acs.est.9b02971

[11] Wei W , Huang Q‐S , Sun J , Wang J‐Y , Wu S‐L , Ni B‐J . Polyvinyl chloride microplastics affect methane production from the anaerobic digestion of waste‐activated sludge through leaching toxic Bisphenol‐A. Environ Sci Technol. 2019;53:2509–2517. 30758964 10.1021/acs.est.8b07069

[12] Fu S‐F , Ding J‐N , Zhang Y , Li Y‐F , Zhu R , Yuan X‐Z , et al. Exposure to polystyrene nanoplastic leads to inhibition of anaerobic digestion system. Sci Total Environ. 2018;625:64–70. 29289007 10.1016/j.scitotenv.2017.12.158

[13] Feng L‐J , Wang J‐J , Liu S‐C , Sun X‐D , Yuan X‐Z , Wang S‐G . Role of extracellular polymeric substances in the acute inhibition of activated sludge by polystyrene nanoparticles. Environ Pollut. 2018;238:859–865. 29627756 10.1016/j.envpol.2018.03.101

[14] Bardgett RD , Caruso T . Soil microbial community responses to climate extremes: resistance, resilience and transitions to alternative states. Philos Trans R Soc B. 2020;375:20190112. 10.1098/rstb.2019.0112 PMC7017770 31983338

[15] Xu G , He J . Resilience of organohalide‐detoxifying microbial community to oxygen stress in sewage sludge. Water Res. 2022;224:119055. 36126627 10.1016/j.watres.2022.119055

[16] Li W , Khalid H , Zhu Z , Zhang R , Liu G , Chen C , et al. Methane production through anaerobic digestion: participation and digestion characteristics of cellulose, hemicellulose and lignin. Appl Energy. 2018;226:1219–1228.

[17] Yu J , Tang SN , Lee PKH . Microbial communities in full‐scale wastewater treatment systems exhibit deterministic assembly processes and functional dependency over time. Environ Sci Technol. 2021;55:5312–5323. 33784458 10.1021/acs.est.0c06732

[18] Vanwonterghem I , Jensen PD , Dennis PG , Hugenholtz P , Rabaey K , Tyson GW . Deterministic processes guide long‐term synchronised population dynamics in replicate anaerobic digesters. ISME J. 2014;8:2015–2028. 24739627 10.1038/ismej.2014.50 PMC4184015

[19] Peces M , Astals S , Jensen PD , Clarke WP . Deterministic mechanisms define the long‐term anaerobic digestion microbiome and its functionality regardless of the initial microbial community. Water Res. 2018;141:366–376. 29807319 10.1016/j.watres.2018.05.028

[20] Liu J , Xu G , Zhao S , Chen C , Rogers MJ , He J . Mechanistic and microbial ecological insights into the impacts of micro‐ and nano‐ plastics on microbial reductive dehalogenation of organohalide pollutants. J Hazard Mater. 2023;448:130895. 36758435 10.1016/j.jhazmat.2023.130895

[21] Zettler ER , Mincer TJ , Amaral‐Zettler LA . Life in the “plastisphere”: microbial communities on plastic marine debris. Environ Sci Technol. 2013;47:7137–7146. 23745679 10.1021/es401288x

[22] Amaral‐Zettler LA , Zettler ER , Mincer TJ . Ecology of the plastisphere. Nat Rev Microbiol. 2020;18:139–151. 31937947 10.1038/s41579-019-0308-0

[23] Cornejo‐D'Ottone M , Molina V , Pavez J , Silva N . Greenhouse gas cycling by the plastisphere: the sleeper issue of plastic pollution. Chemosphere. 2020;246:125709. 31901660 10.1016/j.chemosphere.2019.125709

[24] Su X , Yang L , Yang K , Tang Y , Wen T , Wang Y , et al. Estuarine plastisphere as an overlooked source of N_2_O production. Nat Commun. 2022;13:3884. 35794126 10.1038/s41467-022-31584-x PMC9259610

[25] Yuan MM , Guo X , Wu L , Zhang Y , Xiao N , Ning D , et al. Climate warming enhances microbial network complexity and stability. Nat Clim Change. 2021;11:343–348.

[26] Zhu D , Ma J , Li G , Rillig MC , Zhu Y‐G . Soil plastispheres as hotspots of antibiotic resistance genes and potential pathogens. ISME J. 2022;16:521–532. 34455424 10.1038/s41396-021-01103-9 PMC8776808

[27] Rosato A , Barone M , Negroni A , Brigidi P , Fava F , Xu P , et al. Microbial colonization of different microplastic types and biotransformation of sorbed PCBs by a marine anaerobic bacterial community. Sci Total Environ. 2020;705:135790. 31972939 10.1016/j.scitotenv.2019.135790

[28] Hernandez DJ , David AS , Menges ES , Searcy CA , Afkhami ME . Environmental stress destabilizes microbial networks. ISME J. 2021;15:1722–1734. 33452480 10.1038/s41396-020-00882-x PMC8163744

[29] Nemergut DR , Schmidt SK , Fukami T , O'Neill SP , Bilinski TM , Stanish LF , et al. Patterns and processes of microbial community assembly. Microbiol Mol Biol Rev. 2013;77:342–356. 24006468 10.1128/MMBR.00051-12 PMC3811611

[30] Rillig MC , Leifheit E , Lehmann J . Microplastic effects on carbon cycling processes in soils. PLoS Biol. 2021;19:e3001130. 33784293 10.1371/journal.pbio.3001130 PMC8009438

[31] Seeley ME , Song B , Passie R , Hale RC . Microplastics affect sedimentary microbial communities and nitrogen cycling. Nat Commun. 2020;11:2372. 32398678 10.1038/s41467-020-16235-3 PMC7217880

[32] He Z‐W , Yang W‐J , Ren Y‐X , Jin H‐Y , Tang C‐C , Liu W‐Z , et al. Occurrence, effect, and fate of residual microplastics in anaerobic digestion of waste activated sludge: a state‐of‐the‐art review. Bioresour Technol. 2021;331:125035. 33820702 10.1016/j.biortech.2021.125035

[33] Hu M , Palić D . Micro‐ and nanoplastics activation of oxidative and inflammatory adverse outcome pathways. Redox Biol. 2020;37:101620. 32863185 10.1016/j.redox.2020.101620 PMC7767742

[34] Wang Y , Wang S , Xu T , Cui W , Shi X , Xu S . A new discovery of polystyrene microplastics toxicity: the injury difference on bladder epithelium of mice is correlated with the size of exposed particles. Sci Total Environ. 2022;821:153413. 35090911 10.1016/j.scitotenv.2022.153413

[35] Yin L , Chen B , Xia B , Shi X , Qu K . Polystyrene microplastics alter the behavior, energy reserve and nutritional composition of marine jacopever (*Sebastes schlegelii*). J Hazard Mater. 2018;360:97–105. 30098534 10.1016/j.jhazmat.2018.07.110

[36] Sun X , Chen B , Li Q , Liu N , Xia B , Zhu L , et al. Toxicities of polystyrene nano‐ and microplastics toward marine bacterium *Halomonas alkaliphila* . Sci Total Environ. 2018;642:1378–1385. 30045518 10.1016/j.scitotenv.2018.06.141

[37] Matthews S , Mai L , Jeong C‐B , Lee J‐S , Zeng EY , Xu EG . Key mechanisms of micro‐ and nanoplastic (MNP) toxicity across taxonomic groups. Comp Biochem Physiol Part C Toxicol Pharmacol. 2021;247:109056. 10.1016/j.cbpc.2021.109056 33894368

[38] Liang Z , Xu G , Shi J , Yu S , Lu Q , Liang D , et al. Sludge digestibility and functionally active microorganisms in methanogenic sludge digesters revealed by *E. coli*‐fed digestion and microbial source tracking. Environ Res. 2021;193:110539. 33253703 10.1016/j.envres.2020.110539

[39] Coyte KZ , Schluter J , Foster KR . The ecology of the microbiome: networks, competition, and stability. Science. 2015;350:663–666. 26542567 10.1126/science.aad2602

[40] Chen S , He J , Wang H , Dong B , Li N , Dai X . Microbial responses and metabolic pathways reveal the recovery mechanism of an anaerobic digestion system subjected to progressive inhibition by ammonia. Chem Eng J. 2018;350:312–323.

[41] Khafipour A , Jordaan EM , Flores‐Orozco D , Khafipour E , Levin DB , Sparling R , et al. Response of microbial community to induced failure of anaerobic digesters through overloading with propionic acid followed by process recovery. Front Bioeng Biotechnol. 2020;8:1–13. 33363133 10.3389/fbioe.2020.604838 PMC7759631

[42] De Vrieze J , Christiaens MER , Walraedt D , Devooght A , Ijaz UZ , Boon N . Microbial community redundancy in anaerobic digestion drives process recovery after salinity exposure. Water Res. 2017;111:109–117. 28063283 10.1016/j.watres.2016.12.042

[43] Allison SD , Martiny JBH . Resistance, resilience, and redundancy in microbial communities. Proc Natl Acad Sci USA. 2008;105:11512–11519. 18695234 10.1073/pnas.0801925105 PMC2556421

[44] Niu Q , Takemura Y , Kubota K , Li Y‐Y . Comparing mesophilic and thermophilic anaerobic digestion of chicken manure: microbial community dynamics and process resilience. Waste Manage. 2015;43:114–122. 10.1016/j.wasman.2015.05.012 26054964

[45] Carballa M , Regueiro L , Lema JM . Microbial management of anaerobic digestion: exploiting the microbiome‐functionality nexus. Curr Opin Biotechnol. 2015;33:103–111. 25682574 10.1016/j.copbio.2015.01.008

[46] Chen Z , Gu T , Wang X , Wu X , Sun J . Oxygen gradients shape the unique structure of picoeukaryotic communities in the Bay of Bengal. Sci Total Environ. 2022;814:152862. 35016938 10.1016/j.scitotenv.2021.152862

[47] Wang J , Qin X , Guo J , Jia W , Wang Q , Zhang M , et al. Evidence of selective enrichment of bacterial assemblages and antibiotic‐resistant genes by microplastics in urban rivers. Water Res. 2020;183:116113. 32668354 10.1016/j.watres.2020.116113

[48] Lu Q , Yu Z , Yu S , Liang Z , Li H , Sun L , et al. Organic matter rather than salinity as a predominant feature changes performance and microbiome in methanogenic sludge digesters. J Hazard Mater. 2019;377:349–356. 31173985 10.1016/j.jhazmat.2019.05.075

[49] Ding C , Rogers MJ , He J . *Dehalococcoides mccartyi* strain GEO12 has a natural tolerance to chloroform inhibition. Environ Sci Technol. 2020;54:8750–8759. 32551613 10.1021/acs.est.0c00993

[50] Xu G , Lu Q , Yu L , Wang S . Tetrachloroethene primes reductive dechlorination of polychlorinated biphenyls in a river sediment microcosm. Water Res. 2019;152:87–95. 30665163 10.1016/j.watres.2018.12.061

[51] Bolyen E , Rideout JR , Dillon MR , Bokulich NA , Abnet CC , Al‐Ghalith GA , et al. Reproducible, interactive, scalable and extensible microbiome data science using QIIME 2. Nat Biotechnol. 2019;37:852–857. 31341288 10.1038/s41587-019-0209-9 PMC7015180

[52] Callahan BJ , McMurdie PJ , Rosen MJ , Han AW , Johnson AJA , Holmes SP . DADA2: high‐resolution sample inference from Illumina amplicon data. Nat Methods. 2016;13:581–583. 27214047 10.1038/nmeth.3869 PMC4927377

[53] Stamatakis A . RAxML version 8: a tool for phylogenetic analysis and post‐analysis of large phylogenies. Bioinformatics. 2014;30:1312–1313. 24451623 10.1093/bioinformatics/btu033 PMC3998144

[54] Katoh K , Misawa K , Kuma K , Miyata T . MAFFT: a novel method for rapid multiple sequence alignment based on fast Fourier transform. Nucleic Acids Res. 2002;30:3059–3066. 12136088 10.1093/nar/gkf436 PMC135756

[55] Bokulich NA , Kaehler BD , Rideout JR , Dillon M , Bolyen E , Knight R , et al. Optimizing taxonomic classification of marker‐gene amplicon sequences with QIIME 2's q2‐feature‐classifier plugin. Microbiome. 2018;6:90. 29773078 10.1186/s40168-018-0470-z PMC5956843

[56] Quast C , Pruesse E , Yilmaz P , Gerken J , Schweer T , Yarza P , et al. The SILVA ribosomal RNA gene database project: improved data processing and web‐based tools. Nucleic Acids Res. 2012;41:D590–D596. 23193283 10.1093/nar/gks1219 PMC3531112

[57] Chen EX , Qiu M , Zhang YF , Zhu YS , Liu LY , Sun YY , et al. Acid and base‐resistant zirconium polyphenolate‐metalloporphyrin scaffolds for efficient CO_2_ photoreduction. Adv Mater. 2018;30:1–8. 10.1002/adma.201704388 29178432

[58] Bastian M , Heymann S , Jacomy M . Gephi: an open source software for exploring and manipulating networks. In *Third International ICWSM Conference* 2009: 361–362.

[59] Rice EW , Bridgewater L , American Public Health Association . *Standard methods for the examination of water and wastewater* . Vol. 10. Washington, DC: American Public Health Association; 2012.

