# Supplementary material for: Resilience and functional redundancy of methanogenic digestion microbiome safeguard recovery of methanogenesis activity under the stress induced by microplastics
Source: mLife. 2023 Dec 15;2(4):378–88. doi: 10.1002/mlf2.12090 (PMC10989149; doi:10.1002/mlf2.12090)
Supplement: Supplementary file 1 — Supporting information. [file MLF2-2-378-s001.docx]

**Supporting information for**

**Resilience and Functional Redundancy of Methanogenic Digestion Microbiome Safeguard Recovery of Methanogenesis Activity under the Stress Induced by Microplastics**

Jinting Liu, Guofang Xu, Siyan Zhao, Jianzhong He^*^

Department of Civil and Environmental Engineering, National University of Singapore, Singapore 117576

**^*^**Corresponding author:

Department of Civil and Environmental Engineering, National University of Singapore, Block E2-02-13, 1 Engineering Drive 3, Singapore 117576, Singapore

Email address: [jianzhong.he@nus.edu.sg](mailto:jianzhong.he@nus.edu.sg)

Number of pages: 14; Number of methods: 5; Number of figures: 7; Number of tables: 3

**Method S1: Measurement of reactive oxygen species (ROS)**

ROS was measured using the 2’7’-dichlorodihydrofluorescein diacetate (H_2_DCF-DA) method.^1^ Briefly, 2 mL samples containing plastics were centrifuged at 15000 g for 15 min, then the pellets were washed using 0.1 mol/L phosphate buffer and resuspended in buffer added with 50 μmol H_2_DCF-DA and incubated in dark at 35°C. The H_2_DCF-DA was oxidized to fluorescent dichlorofluorescein (DCF) by ROS. Then, the pellets were centrifuged at 15000g for 15 min and placed into a 96-well plate with phosphate buffer. DCF was measured using a fluorescence spectroscopy with an excitation wavelength of 485 nm and an emission wavelength of 520 nm.^2^

**Method S2: Methane gas production calculation**

The volume of biogas generated during the sludge anaerobic digestion was estimated by releasing the pressure to equilibrate with the room pressure using a glass syringe (250 mL). The amount of methane gas was computed as follows.^3^

$$V_{Methane,i}=C_{Methane,i}\times V_{G,i}-C_{Methane,i-1}\times V_{G,i-1}$$

where V*_Methane,i_* is the volume of methane gas; V_G,i_ and V_G,i-1_ are the total gas volumes in the current and previous sampling time points, respectively; and C*_Metahne,i_* and C*_Methane,i-1_* are the fractions of methane gas measured by gas chromatography in the current and previous sampling time points, respectively.

**Method S3: EPS extraction**

The sludge sample was first centrifuged at 1500 g for 15 min and then the residual pellet was re-suspended to its original volume by adding 0.05% NaCl solution. The solution was then heated at 60 °C for 30 min, and finally centrifuged at 15000g for 15 min. The supernatant was filtered through a 0.45 μm membrane, and the filtrate was subjected to measurement of EPS.

**Method S4: Determination of plastic amendment concentration**

The concentration of plastics amended into the sludge was determined by a two-step approach, combining theoretical calculations and microscopic validation. Briefly, theoretical calculations were performed based on the plastic diameter and density provided by the manufacturers. The plastics were assumed to be spherical in shape. The volume of plastic particle was determined using the formula for the volume of a sphere based on the diameter. The mass per particle was then calculated based the volume and density of the plastics. To further confirm the final concentrations of plastics in sludge, experimental validation was carried out using microscope (Fig.S6).

**Method S5: Determination of plastic concentration in taw WAS and DS**

The microplastic concentration in raw WAS and DS were determined according to the reported method.^4^ Briefly, 50 g of WAS/DS was firstly mixed with 750 mL saturated sodium chloride. The mixture was then stirred for 10 min and settled for 3 h. The supernatant was sieved with 10 μm metal mesh. The residues were washed with distilled water and treated with 30% hydrogen peroxide solution overnight. The supernatants were filtered through a nitrate cellulose membrane (0.45 µm; 47-mm Ø, Advantec, Japan) to capture the target microplastics. The extracted plastic samples were then washed, dried, and examined under microscope and visually identified (Fig.S5). All analyses were performed in triplicates.


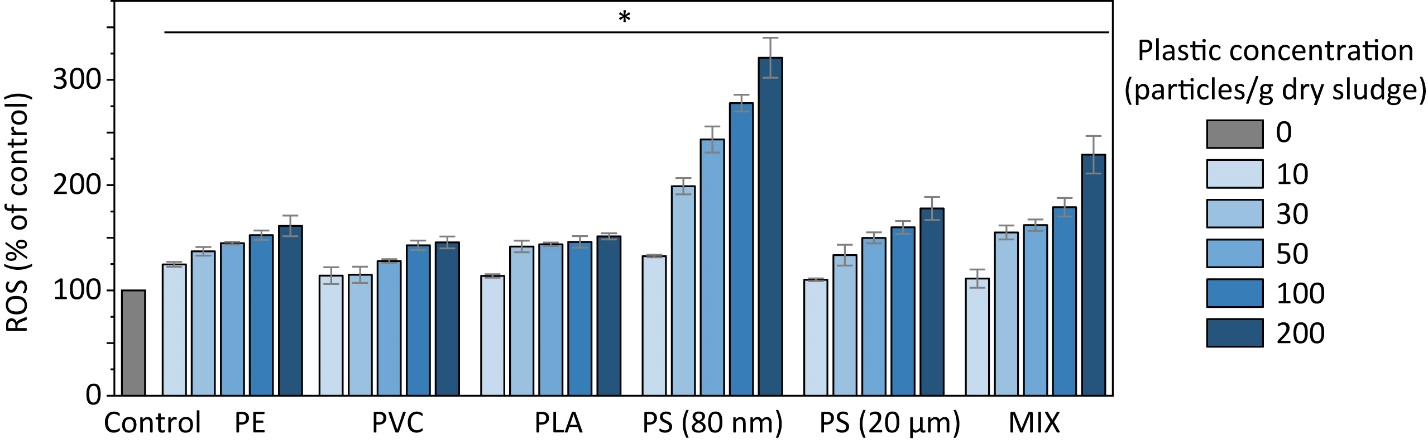


**Figure S1** Relative reactive oxygen species (ROS) production in batch digesters. The values were measured on day 3. Asterisks indicate statistically significant differences compared with the control at the level of *p* = 0.05 based on two-sample t-test.


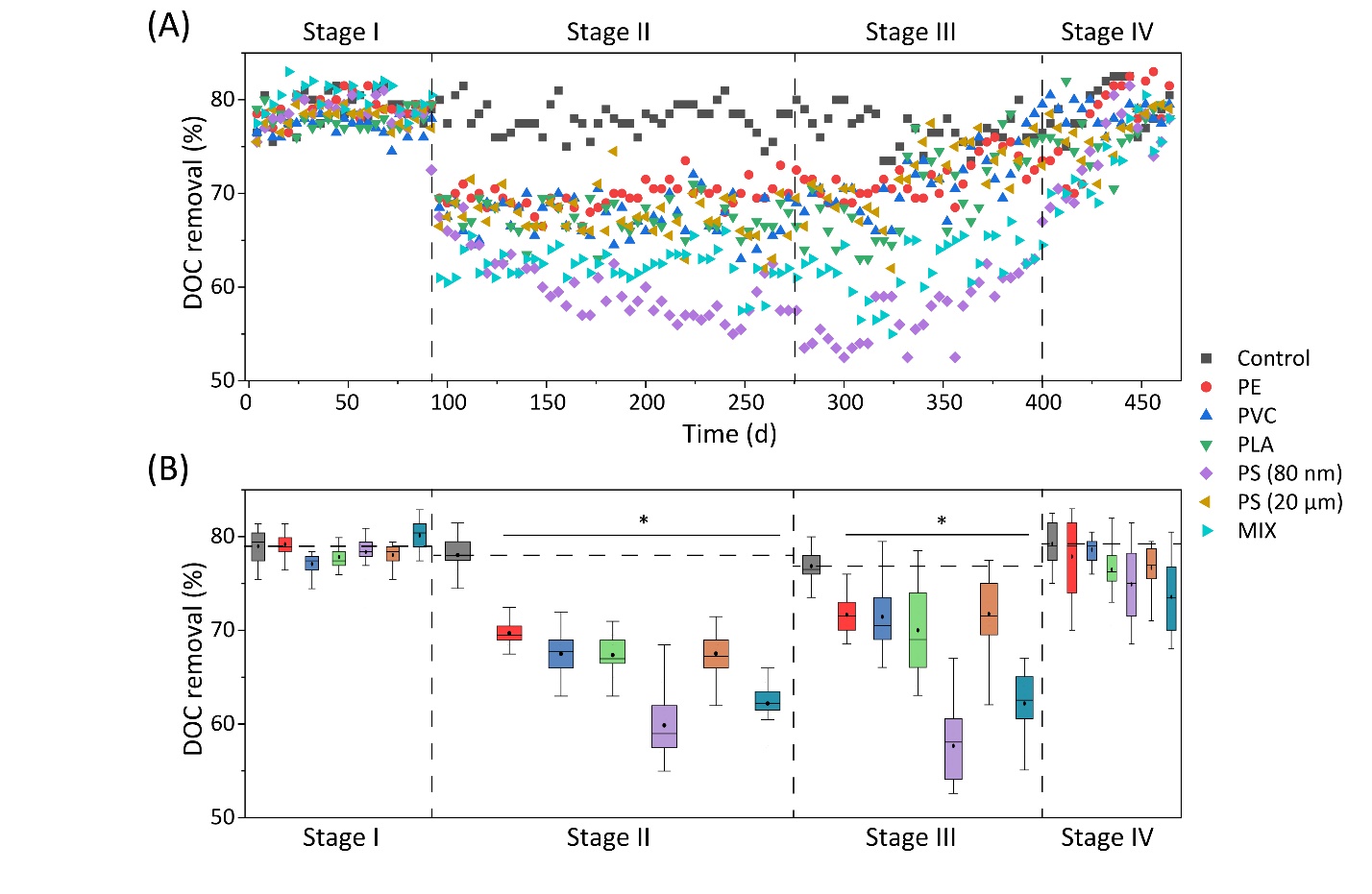


**Figure S2** Removal of DOC in semicontinuous methanogenic digesters. Asterisks indicate statistically significant differences compared with the control at the level of *p* = 0.05 based on two-sample t-test.

**
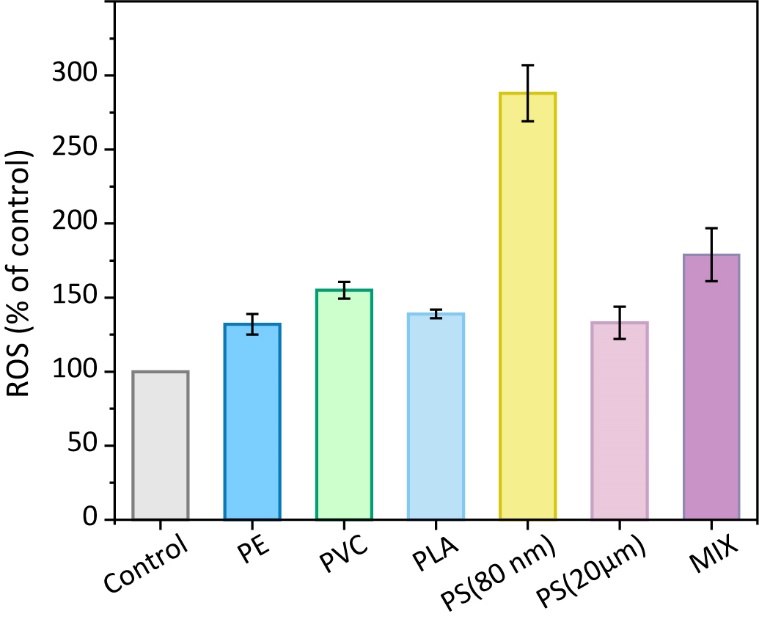
**

**Figure S3** Relative reactive oxygen species (ROS) production in semicontinuous methanogenic digesters at stage-IV.


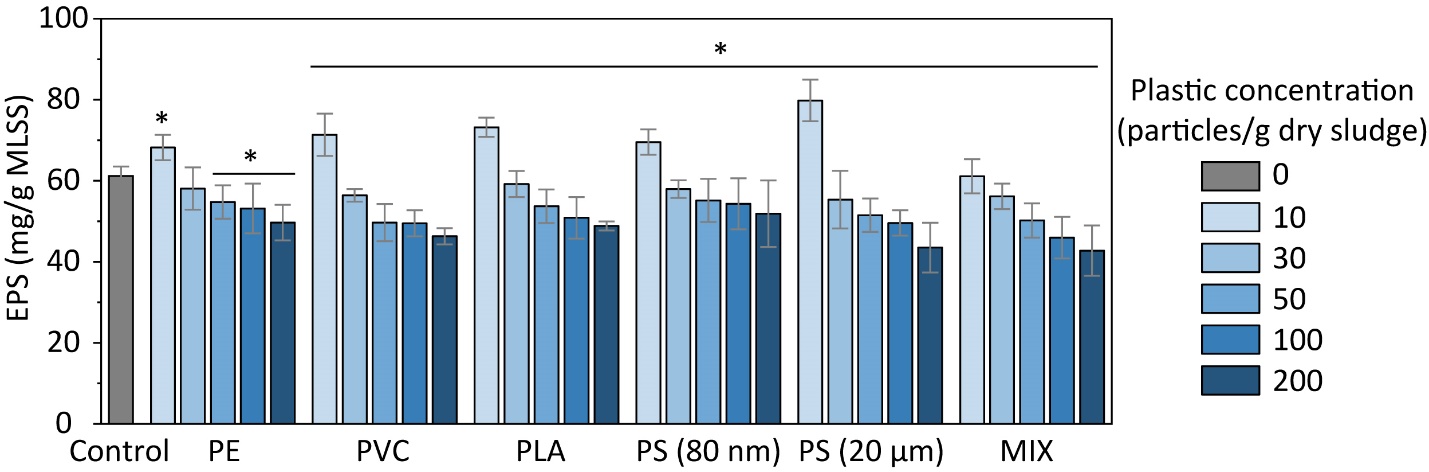


**Figure S4** The concentration of EPS in batch digesters. The values were measured on day 3. Asterisks indicate statistically significant differences compared with the control at the level of *p* = 0.05 based on two-sample t-test.

**
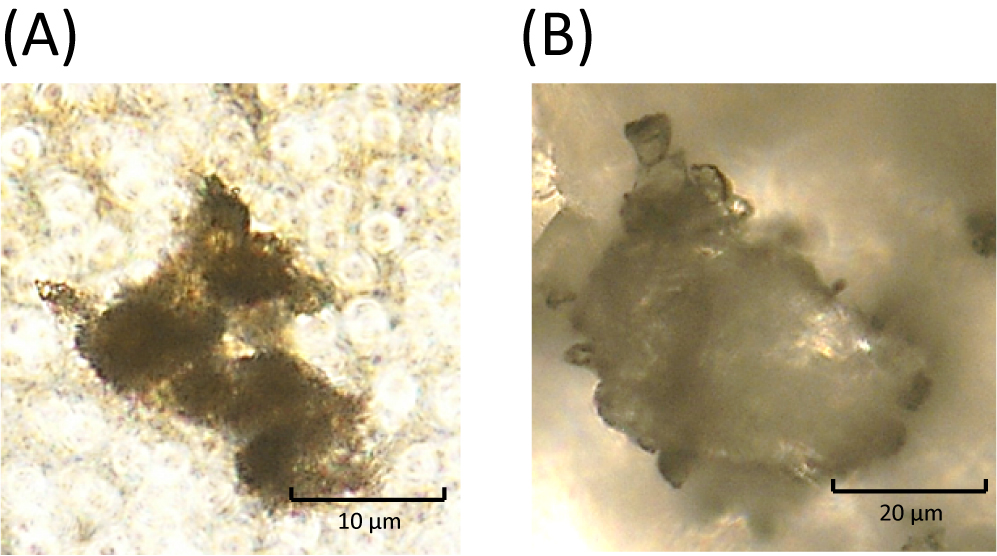
**

**Figure S5** Microscopic analysis of microplastics in raw (A) WAS and (B) DS.


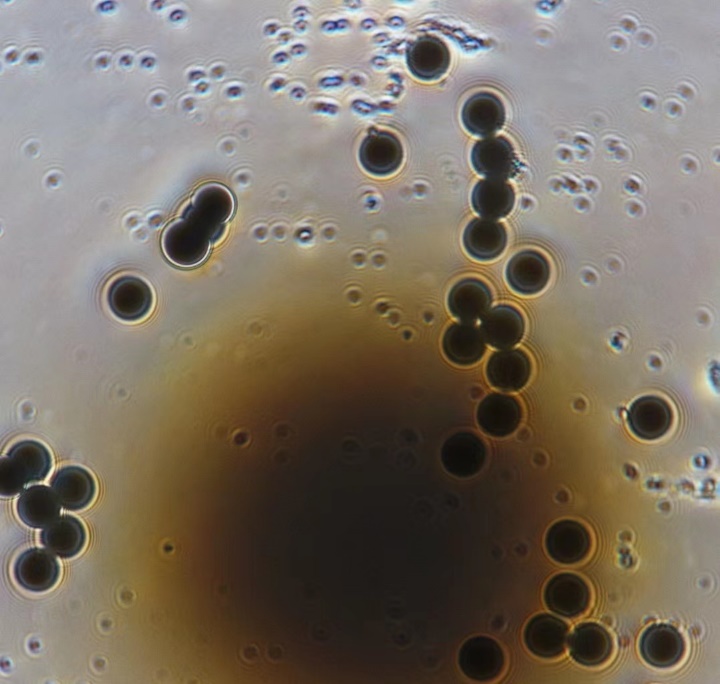


**Figure S6** Microscopic analysis for plastic quantification.

**
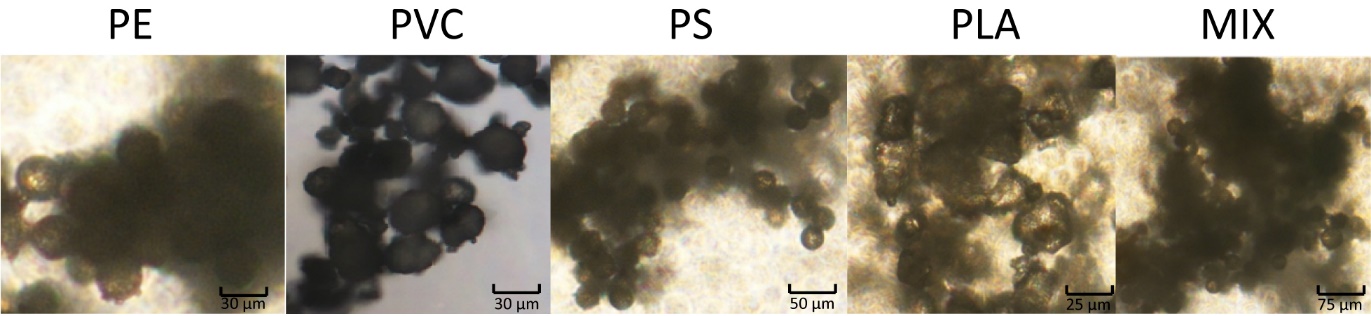
**

**Figure S7** Microscopic analysis of plastics collected from sludge.

**Table S1** Properties of DS, WAS, and thermal pre-treated WAS.

| Parameters | DS | WAS | Thermal pre-treated WAS |
| --- | --- | --- | --- |
| Total solids (TS; g/L) | 22.1 ± 0.1 | 35.6 ± 0.3 | 19.1 ± 0.5 |
| Volatile solids (VS; g/L) | 16.7 ± 0.2 | 25.4 ± 0.2 | 13.6 ± 0.2 |
| Total chemical oxygen demand (TCOD; g/L) | 22.4 ± 0.2 | 40.2 ± 0.4 | 45.6 ± 0.3 |
| Soluble chemical oxygen demand (SCOD; g/L) | 3.3 ± 0.1 | 3.6 ± 0.1 | 18.9 ± 0.3 |
| pH | 7.6 ± 0.1 | 7.0 ± 0.1 | 6.9 ± 0.1 |

**Table S2** PERMANOVA analysis of digestion sludge and the plastisphere microbiome.

|  |  | Sludge | | | | | | | Plastisphere | | | | | |
| --- | --- | --- | --- | --- | --- | --- | --- | --- | --- | --- | --- | --- | --- | --- |
|  |  | Control | PE | PVC | PS (20μm) | PS (80 nm) | PLA | MIX | PE | PVC | PS (20μm) | PS (80 nm) | PLA | MIX |
| Sludge | Control | / | 0.7 | 0.24 | 0.445 | 0.074 | 0.131 | 0.53 | 0.002 | 0.004 | 0.002 | 0.004 | 0.048 | 0.001 |
|  | PE |  | / | 0.162 | 0.752 | 0.183 | 0.303 | 0.802 | 0.001 | 0.01 | 0.005 | 0.027 | 0.193 | 0.006 |
|  | PVC |  |  | / | 0.061 | 0.001 | 0.025 | 0.051 | 0.001 | 0.001 | 0.001 | 0.001 | 0.053 | 0.001 |
|  | PS (20μm) |  |  |  | / | 0.122 | 0.217 | 0.699 | 0.001 | 0.003 | 0.001 | 0.009 | 0.021 | 0.002 |
|  | PS (80 nm) |  |  |  |  | / | 0.779 | 0.015 | 0.001 | 0.001 | 0.001 | 0.004 | 0.001 | 0.001 |
|  | PLA |  |  |  |  |  | / | 0.054 | 0.001 | 0.001 | 0.001 | 0.002 | 0.009 | 0.054 |
|  | MIX |  |  |  |  |  |  | / | 0.001 | 0.003 | 0.001 | 0.003 | 0.024 | 0.001 |
| Plastisphere | PE |  |  |  |  |  |  |  | / | 0.15 | 0.038 | 0.067 | 0.001 | 0.098 |
|  | PVC |  |  |  |  |  |  |  |  | / | 0.636 | 0.604 | 0.031 | 0.415 |
|  | PS (20μm) |  |  |  |  |  |  |  |  |  | / | 0.683 | 0.029 | 0.591 |
|  | PS (80 nm) |  |  |  |  |  |  |  |  |  |  | / | 0.075 | 0.23 |
|  | PLA |  |  |  |  |  |  |  |  |  |  |  | / | 0.011 |
|  | MIX |  |  |  |  |  |  |  |  |  |  |  |  | / |

**Table S3** Network properties of digestion sludge and the plastisphere microbiome.

|  | Samples | Number of nodes | Number of edges | Average degree | Density | Modularity | Degree centralization | Positive interactions ratio |
| --- | --- | --- | --- | --- | --- | --- | --- | --- |
| Sludge | Control | 413 | 1809 | 8.76 | 0.021 | 1.218 | 0.513 | 74.07 |
|  | PE | 451 | 3257 | 14.443 | 0.032 | 3.961 | 0.554 | 56.37 |
|  | PVC | 515 | 3056 | 11.868 | 0.023 | 1.94 | 0.474 | 61.65 |
|  | PLA | 496 | 3096 | 12.484 | 0.025 | 1.735 | 0.53 | 66.51 |
|  | PS (0.08) | 510 | 3124 | 12.251 | 0.024 | 4.232 | 0.51 | 55.51 |
|  | PS (20) | 494 | 2988 | 12.097 | 0.025 | 2.109 | 0.542 | 62.35 |
|  | MIX | 467 | 2524 | 10.809 | 0.023 | 2.036 | 0.503 | 63.15 |
| Plastisphere | PE | 392 | 1226 | 6.255 | 0.016 | 1.253 | 0.512 | 75.45 |
|  | PVC | 434 | 1477 | 6.806 | 0.016 | 4.75 | 0.504 | 55.18 |
|  | PLA | 493 | 2298 | 9.323 | 0.019 | 2.385 | 0.499 | 60.23 |
|  | PS (0.08) | 463 | 1849 | 7.987 | 0.017 | 5.014 | 0.517 | 55.00 |
|  | PS (20) | 434 | 1645 | 7.581 | 0.018 | 2.373 | 0.534 | 61.34 |
|  | MIX | 354 | 1516 | 8.565 | 0.024 | 1.278 | 0.509 | 71.97 |

**Reference:**

1. Limbach, L. K.; Wick, P.; Manser, P.; Grass, R. N.; Bruinink, A.; Stark, W. J., Exposure of Engineered Nanoparticles to Human Lung Epithelial Cells:  Influence of Chemical Composition and Catalytic Activity on Oxidative Stress. *Environ Sci Technol* **2007,** *41*, (11), 4158-4163.

2. Jeong, C.-B.; Won, E.-J.; Kang, H.-M.; Lee, M.-C.; Hwang, D.-S.; Hwang, U.-K., et al., Microplastic Size-Dependent Toxicity, Oxidative Stress Induction, and p-JNK and p-p38 Activation in the Monogonont Rotifer (Brachionus koreanus). *Environ Sci Technol* **2016,** *50*, (16), 8849-8857.

3. Zhang, B.; Tang, X.; Fan, C.; Hao, W.; Zhao, Y.; Zeng, Y., Cationic polyacrylamide alleviated the inhibitory impact of ZnO nanoparticles on anaerobic digestion of waste activated sludge through reducing reactive oxygen species induced. *Water Res.* **2021,** *205*, 117651.

4. Li, X.; Chen, L.; Mei, Q.; Dong, B.; Dai, X.; Ding, G., et al., Microplastics in sewage sludge from the wastewater treatment plants in China. *Water Research* **2018,** *142*, 75-85.
